# Examining the dimensions and correlates of workplace stress among Australian veterinarians

**DOI:** 10.1186/1745-6673-4-32

**Published:** 2009-12-08

**Authors:** Derek R Smith, Peter A Leggat, Richard Speare, Maureen Townley-Jones

**Affiliations:** 1 WorkCover New South Wales Research Centre of Excellence, School of Health Sciences, Faculty of Health, University of Newcastle, Ourimbah, 2258, Australia; 2 Anton Breinl Centre for Public Health and Tropical Medicine, James Cook University, Townsville, 4811, Australia; 3 School of Mathematical and Physical Sciences, Faculty of Science and Information Technology, University of Newcastle, Ourimbah, 2258, Australia

## Abstract

**Background:**

Although stress is known to be a common occupational health issue in the veterinary profession, few studies have investigated its broad domains or the internal validity of the survey instrument used for assessment.

**Methods:**

We analysed data from over 500 veterinarians in Queensland, Australia, who were surveyed during 2006-07.

**Results:**

The most common causes of stress were reported to be long hours worked per day, not having enough holidays per year, not having enough rest breaks per day, the attitude of customers, lack of recognition from the public and not having enough time per patient. Age, gender and practice type were statistically associated with various aspects of work-related stress. Strong correlations were found between having too many patients per day and not having enough time per patient; between not having enough holidays and long working hours; and also between not enough rest breaks per day and long working hours. Factor analysis revealed four dimensions of stress comprising a mixture of career, professional and practice-related items. The internal validity of our stress questionnaire was shown to be high during statistical analysis.

**Conclusion:**

Overall, this study suggests that workplace stress is fairly common among Australian veterinarians and represents an issue that occupies several distinct areas within their professional life.

## Background

Veterinarians are exposed to a wide variety of occupational hazards during their working life, including bites, scratches and other trauma from animals, needlestick and sharps injuries, musculoskeletal disorders, occupational dermatoses, car accidents, as well as exposures to zoonotic diseases, x-rays, anaesthetic gases and other chemicals [1-10]. In recent years, increasing attention has been paid to psychosocial factors and work stress among veterinarians, including client interaction, career structure, peer support and suicide [11-18]. A longitudinal study of Australian veterinarians, for example, found that work stress was an important concern for many after 10 years in practice [19]. Stress is not evenly distributed, however, with gender, age and practice type known to be important correlates [12,20].

Despite this fact, only a few studies have specifically looked at stress among Australian veterinarians, and even fewer have conducted a detailed analysis of stress domains and the associated validity of the survey instrument used for assessment. The purpose of our current study therefore, was to analyse the dimensions of stress among Australian veterinarians, to establish whether certain psychosocial factors were influencing the development and severity of their symptoms, and to assess the statistical validity of our survey instrument.

## Methods

An anonymous questionnaire survey was mailed to all veterinarians who were registered with the *Veterinary Surgeons Board of Queensland *and included on the veterinary roll for 2006 [21]. This method was chosen to ensure maximum cost-effectiveness and minimum disruption to their working lives. Our survey instrument was based on previous investigations conducted in the veterinary profession [11,12,19], and requested information on demographic and workplace items such as age, sex, weekly working hours, practice type and total career length. Information was then sought regarding perceived stress levels. No exact definition of stress was provided, similar to a previous study from New Zealand [12], so that respondents could use their own interpretation of what 'stress' actually meant to them. Questions were grouped into three stress dimensions, encompassing 6 topics each: [1] *Career Factors *(career structure, promotion, salary, work hours, rest breaks and holidays), [2] *Professional Factors *(attitude of colleagues/workmates/superiors/customers, recognition from the public/colleagues or family) and [3] *Practice-Related Issues *(number of patients per day, pressure to over service, the possibility of litigation, potential danger from animals/diseases). Responses were collated on a five-way Likert-type scale [22], ranging from 'none' to 'extreme'.

Questionnaires were mailed to all veterinarians during 2006, with follow-up reminders sent to all participants who had not responded to either the first or second mailing. Data was anonymously entered into a spreadsheet program and statistically analysed. Factor analysis (principal component method and varimax rotation) was performed for all 18 items of the stress questionnaire. Factors with Eigen Values greater than 1 were extracted. The internal consistency of the stress scale was ascertained by calculating Cronbach's alpha [23,24].

## Results

Surveys were distributed to 1038 eligible participants, from whom 664 were returned, giving a response rate of 64.0%. Participants with incomplete or missing answers were then excluded, leaving a total of 567 veterinarians for the final analysis. Slightly less than half (45%) were female. Around one-third (32%) were aged over 50 years, with 31-40 years the second largest age range (comprising 28% of the respondents). Approximately half (48%) worked 31-50 hours per week, 42% treated over 50 patients per week and 53% were their own principal employer. Slightly less than half the respondents (47%) worked in small animal practice.

**Figure 1:**
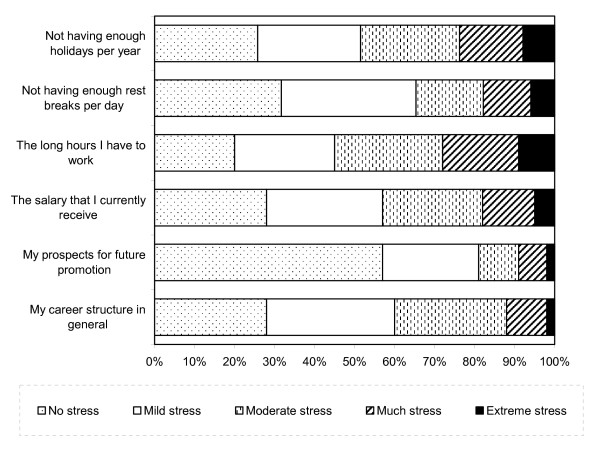
**Likert Scale Responses to Career Factors**.

**Figure 2:**
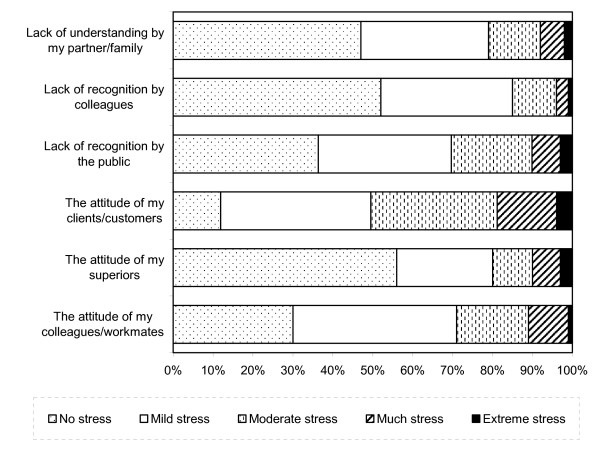
**Likert Scale Responses to Professional Factors**.

**Figure 3:**
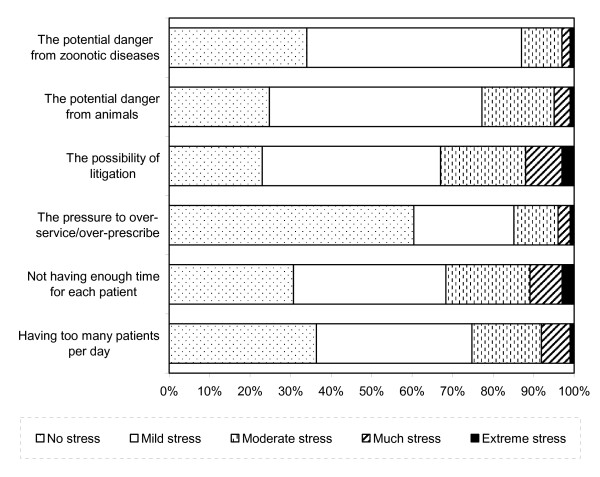
**Likert Scale Responses to Practice Issues**.

Likert Scale Responses to Career Factors are displayed in Figure 1. Long hours worked per day, not having enough holidays per year and not having enough rest breaks per day were the most likely to have caused extreme stress for veterinarians (9%, 8% and 6%, respectively). Prospects for future promotion were the least likely to have caused stress, with over half (57%) of all respondents reporting experiencing no stress from these issues at all. Likert Scale responses to professional factors are displayed in Figure 2. The attitude of clients/customers and a lack of recognition from the public were the most common causes of stress among veterinarians, causing extreme stress among 4% and 3%, respectively. Over half (56%) experienced no stress due to the attitude of their superiors. Likert Scale Responses to Practice Issues are displayed in Figure 3. One-in-ten veterinarians reported experiencing considerable or extreme stress due to not having enough time per patient. A similar proportion also reported extreme stress related to the possibility of litigation. Almost two-thirds (61%) experienced no stress from pressure to over-service or over-prescribe.

Demographic and work-related correlations with veterinary stress questions are displayed in Table 1. Age showed significant correlation with stress due to prospects for future promotion, current salary and the attitude of superiors (P = 0.001, 0.011 and 0.001, respectively). When compared to their male counterparts, female veterinarians were significantly more likely to report moderate, considerable or extreme stress related to virtually all stress questions, except for not having enough holidays per year, recognition by colleagues and lack of understanding by partner or family (all P > 0.05). Veterinarians in small animal practice were more likely to experience considerable or extreme stress relating to insufficient time per patient (P = 0.002) and pressure to over service or over prescribe (P = 0.010).

**Table 1:** Demographic and Work-Related Correlations with Veterinary Stress Questions^a^

	Age		Gender		Practice Type	
	
	**χ**^**2**^	P	**χ**^**2**^	P	**χ**^**2**^	P
**Career Factors**						
						
A) My career structure in general	9.422	0.051	27.52	0.006*	6.22	0.183
B) My prospects for future promotion	32.036	0.001*	66.24	0.001*	7.15	0.128
C) Salary that I currently receive	13.096	0.011*	36.57	0.003*	1.52	0.822
D) Long hours I have to work	11.136	0.025*	22.90	0.029*	2.59	0.628
E) Not having enough rest breaks per day	8.959	0.062	33.32	0.001*	1.89	0.756
F) Not having enough holidays per year	1.374	0.849	13.38	0.342	6.30	0.178
**Professional Factors**						
						
G) Attitude of my colleagues/workmates	6.562	0.161	31.82	0.002*	3.475	0.482
H) Attitude of my superiors	51.383	0.001*	79.52	0.001*	4.318	0.365
I) Attitude of my clients/customers	4.005	0.405	31.78	0.002*	0.860	0.930
J) Lack of recognition by the public	4.034	0.402	33.52	0.001*	1.972	0.741
K) Lack of recognition by colleagues	7.253	0.123	18.215	0.109	6.864	0.143
L) Lack of understanding by my partner/family	5.588	0.232	19.58	0.076	9.119	0.058
**Practice Issues**						
						
M) Having too many patients per day	4.056	0.399	39.22	0.001*	5.431	0.366
N) Not having enough time for each patient	5.876	0.209	50.94	0.001*	17.481	0.002*
O) Pressure to over-service/over-prescribe	1.794	0.774	37.97	0.001*	13.344	0.010*
P) Possibility of litigation	8.096	0.088	33.80	0.001*	2.375	0.667
Q) Potential danger from animals	3.514	0.476	29.01	0.004*	5.608	0.230
R) Potential danger from zoonotic diseases	1.570	0.814	23.97	0.021*	1.701	0.790

^a ^Adapted from previous studies of veterinary stress [11,12,19], * Statistically significant differences

A correlation matrix for all work stress questions is displayed in Table 2. The internal validity of this component was high, with a Cronbach's Alpha score of 0.887 when all 18 questions were analysed. Strong correlations were found between not having enough time per patient and having too many patients per day (Correlation Coefficient = 0.752), not enough holidays and long working hours (Correlation Coefficient = 0.683) and not enough rest breaks per day and long working hours (Correlation Coefficient = 0.671). Factor loadings are displayed in Table 3. Four dimensions were extracted which accounted for 67% of the variance. The first factor comprised a mixture of career and practice-related items, long working hours, not enough rest, not enough holidays, having too many patients per day and not having enough time per patient (range: 0.740 to 0.802). The second factor focussed on career-related items such as career structure, future promotion and adequate salary (range: 0.608 to 0.818). The third factor focussed on practice-related items such as fear of litigation, danger from animals and danger from zoonotic diseases (range: 0.693 to 0.827). The fourth factor focussed on professional-related items such as the attitude of colleagues and superiors, and lack of colleague recognition (range: 0.628 to 0.717). The internal validity of these items was high (Cronbach's Alpha = 0.869).

**Table 2:** Correlation Matrix for Work Stress Questions among Australian Veterinarians ^a^

	A	B	C	D	E	F	G	H	I	J	K	L	M	N	O	P	Q	R
A	**1.000**																	
B	0.506	**1.000**																
C	0.479	0.571	**1.000**															
D	0.431	0.221	0.444	**1.000**														
E	0.382	0.270	0.363	0.671	**1.000**													
F	0.435	0.161*	0.367	0.683	0.588	**1.000**												
G	0.393	0.206	0.258	0.346	0.372	0.306	**1.000**											
H	0.377	0.520	0.365	0.211	0.282	0.156*	0.367	**1.000**										
I	0.287	0.128**	0.291	0.417	0.397	0.381	0.395	0.239	**1.000**									
J	0.275	0.222	0.350	0.306	0.328	0.325	0.298	0.217	0.504	**1.000**								
K	0.389	0.329	0.317	0.300	0.361	0.312	0.502	0.432	0.337	0.471	**1.000**							
L	0.293	0.165	0.273	0.288	0.291	0.295	0.281	0.165	0.283	0.201	0.241	**1.000**						
M	0.353	0.149*	0.226	0.507	0.529	0.449	0.308	0.197	0.388	0.272	0.302	0.264	**1.000**					
N	0.321	0.194	0.241	0.484	0.530	0.420	0.312	0.219	0.362	0.271	0.313	0.231	0.752	**1.000**				
O	0.188	0.142*	0.214	0.199	0.196	0.174	0.212	0.169	0.304	0.298	0.221	0.201	0.269	0.351	**1.000**			
P	0.200	0.143*	0.224	0.248	0.238	0.259	0.232	0.193	0.401	0.353	0.255	0.187	0.253	0.316	0.406	**1.000**		
Q	0.167	0.123**	0.235	0.227	0.278	0.275	0.224	0.161*	0.370	0.356	0.237	0.257	0.280	0.217	0.310	0.458	**1.000**	
R	0.248	0.133**	0.217	0.199	0.248	0.243	0.184	0.142*	0.208	0.291	0.279	0.197	0.233	0.168	0.205	0.367	0.534	**1.000**

^a ^Adapted from previous studies of veterinary stress [11,12,19] as indicated on Table 1, All correlations significant at P < 0.0001 except *P < 0.001 and **P < 0.005, Cronbach's Alpha = 0.887

**Table 3:** Factor Loadings for Work Stress Responses among Australian Veterinarians

**Dimensions of Stress**^**a**^	Factor 1	Factor 2	Factor 3	Factor 4
Long working hours	**0.802**	0.316	0.113	0.012
Not enough rest	**0.748**	0.242	0.153	0.169
Not enough holidays	**0.748**	0.267	0.203	-0.033
Too many patients/day	**0.763**	-0.058	0.147	0.301
Not enough time per patient	**0.740**	-0.045	0.096	0.349
Career structure	0.351	**0.608**	0.088	0.281
Future promotion	0.021	**0.818**	0.027	0.263
Adequate salary	0.258	**0.791**	0.171	0.034
Fear of litigation	0.179	0.039	**0.693**	0.184
Danger from animals	0.154	0.068	**0.827**	0.076
Danger from diseases	0.097	0.124	**0.798**	0.062
Colleagues attitude	0.276	0.103	0.117	**0.717**
Superiors attitude	0.003	0.499	0.045	**0.628**
No colleague recognition	0.194	0.224	0.211	**0.708**

^a ^Adapted from previous studies of veterinary stress [11,12,19] as indicated on Table 1, Cronbach's Alpha = 0.869

## Discussion

This article presents one of the first studies to specifically analyse dimensions of stress among Australian veterinarians and the validity of its related questionnaire. Australia occupies an important component of the world veterinary demographic. By 2002 for example, the relative number of veterinary practitioners in this country was higher than for the United States (US), United Kingdom (UK) and Canada [25]. The most common causes of extreme stress among them was shown to be long working hours per day, not having enough holidays per year, not having enough rest breaks per day, the attitude of customers, lack of recognition from the public and not having enough time per patient. Such findings are consistent with some previous research conducted among veterinarians in the Asia-Pacific region. In Australia for example [11], the main stressors relating to working conditions were time-related, including long working hours and having insufficient recreation time. In New Zealand [12], total hours worked were shown to be a main cause of stress. In one German study [20], the probability of intense psychosocial stress was shown to increase as the number of work hours increased. In another investigation from the same country [8], correlations were also demonstrated between a high number of working hours and subsequent stress.

Prospects for future promotion, the attitude of superiors and the pressure to over-service or to over-prescribe were the least likely to have caused stress among our Australian veterinarians when the data was analysed as a group. This was somewhat of an interesting finding because quality of leadership and collaboration with co-workers has been previously demonstrated as a stress correlate in other occupations [26]. In the current study however, having insufficient time per patient and the pressure to over-service or over-prescribe were shown to be significant sources of stress for veterinarians in small animal practice. Although the practitioner-client relationship is a very important component of modern veterinary practice, this topic appears to have been rarely studied. What is known is that clients may generate significant negative emotion among veterinarians [13,14]. In a previous Australian study for example [11], clients who did not pay their accounts were a source of chronic irritation and stress for the veterinary practitioner. In New Zealand, client expectations were shown to be an important stressor, especially for females [12]. Personal relationships are also known to affect this particular working group, with difficulties achieving a work-life balance having been previously demonstrated among veterinarians [27]. Stress relating to the work-home interface has also been demonstrated among physicians [28], a comparable occupational group to veterinarians in many ways.

Three main correlates of stress during the current study included age, gender and practice type. Relationships between stress and age have been previously documented in a variety of studies. In Germany for example [20], veterinarians aged 35-54 years were more likely to experience stress than their older counterparts. Similarly in New Zealand [12], younger veterinarians experienced more stress from personal relationships, while family needs were shown to be a particular stressor for those aged between 35 and 54. Interestingly, another Australian study [11] did not record any age-related correlations. In our current investigation, gender was shown to be a strong correlate for almost all aspects of stress. This is again, similar to the aforementioned New Zealand study [12], where females were significantly more stressed than males regarding hours worked, employer/colleague expectations, client expectations, communication with clients, resources, support from senior staff, professional support and unexpected outcomes. In Germany, female veterinarians engaged in high-risk alcohol consumption more often than their male counterparts, although the latter were more likely to binge drink [20]. In an Australian study of workers who euthanize animals [29], females reported higher mean levels of stress when compared to their male counterparts.

Another key finding in the current study was evidence of a relationship between stress and working in small animal practice, particularly regarding not having enough time per patient and the pressure to over-service or over-prescribe. Interestingly, an investigation of New Zealand veterinarians [12] also documented a stress relationship with practice type, albeit in a slightly different manner. Veterinarians working in large or mixed animal practice were more concerned with after hours work than those in small animal practice, while the responsibility for animals' lives was more of a concern for the latter [12]. In Germany, stress was more common among practice owners and veterinarians working in clinical practice than those working elsewhere [20]. It is reasonable to hypothesise that small practice owners or sole operators may be less inclined, or even able, to take sick leave when they feel stressed. Research among their medical counterparts has already shown, for example, that physicians are known to experience a variety of psychosocial stressors [30], and yet, seldom take sick leave and tend to make less use of primary health care services [31]. Workplace health promotion programs may be useful in this regard, particularly considering that reduced work ability is known to be associated with health and work ability [32].

For these reasons, learning to cope with stress remains a critical area of professional practice. While the current study and others have clearly demonstrated that job stress and mental pressure do affect veterinarians [11,12], various anti-stress skills now exist which may help workplace stress to be dealt with in appropriate ways [33]. Support from partners, family and co-workers is always important, and encouragingly, levels of professional support appear to be increasing. A previous study of Australian veterinarians over the past 5 decades, for example, demonstrated that the average recent graduate has had progressively more opportunities for support from other veterinarians [34]. While certain stress-coping skills can be acquired from books or learnt through counselling, they still need to be practiced to enable integration into everyday life [33]. Some research conducted in non-veterinarians has suggested that short duration Stress Management Training (SMT) may be useful in reducing some aspects of stress, anxiety and self-perceived depression [35]. Such strategies may also be useful in the veterinary profession.

Although the current study has clearly demonstrated the presence of stress in Australian veterinary practice, it is also important to keep our findings in perspective. Despite the potential for a wide array occupational hazards, many find that a career in veterinary science is very rewarding [36]. A longitudinal study of veterinarians by Heath [19] found that after 10 years in practice most participants felt that their career had lived up to expectations and was a great source of satisfaction. Our current study has shown that despite some veterinarians experiencing extreme stress in certain areas, the actual proportion was relatively low, and it is reasonable to hypothesise that their overall levels of stress were not excessive, similar to a previous Australian study [11]. On the other hand, a longitudinal investigation from this country reported that almost three-quarters of veterinarians either agreed or strongly agreed that their veterinary work caused them a significant amount of stress [19]. Either way, the results clearly suggest that stress represents an important issue for Australian veterinarians.

While certain limitations were inherent in the current study, including the reliability of self-reported health measures, our investigation nevertheless provides a detailed analysis of stress dimensions among a large cohort of Australian veterinarians, for what appears to be the first time. Although we achieved a relatively high response rate of 64% using standard methods for postal surveys [37], a mixed-mode methodology such as that described by Wilkins and colleagues [38] may have afforded a higher return rate. All of these measures may be useful for future researchers of veterinarians' health to consider.

## Conclusion

Overall, this study has demonstrated important correlations between workplace stress and various career, professional and practice-related issues. It appears that stress remains fairly common among Australian veterinarians, and one that occupies several distinct areas within their professional life. From a methodological perspective, the internal validity of our 18-item stress questionnaire was also shown to be high during statistical analysis. In light of the current findings, greater attention should now be focussed on workplace stress within the veterinary profession, to help ensure that practitioners can more effectively deal with stressful situations faced in their daily working lives.

## Competing interests

The authors declare that they have no competing interests.

## Authors' contributions

PAL conceived the idea for the study and gathered the data. MTJ and DRS performed the statistical design and analysis. DRS, PAL, RS and MTJ drafted the manuscript. All authors read and approved the manuscript.

## References

[B1] Fritschi L Day L Shirangi A Robertson I Lucas M Vizard A Injury in Australian veterinarians Occup Med (Lond) 2006 56 3 199 203 10.1093/occmed/kqj037 16492680

[B2] Jeyaretnam J Jones H Physical, chemical and biological hazards in veterinary practice Aust Vet J 2000 78 11 751 758 10.1111/j.1751-0813.2000.tb10446.x 11194720

[B3] Jeyaretnam J Jones H Phillips M Disease and injury among veterinarians Aust Vet J 2000 78 9 625 629 10.1111/j.1751-0813.2000.tb11939.x 11022291

[B4] Nienhaus A Skudlik C Seidler A Work-related accidents and occupational diseases in veterinarians and their staff Int Arch Occup Environ Health 2005 78 3 230 238 10.1007/s00420-004-0583-5 15776262

[B5] Shirangi A Fritschi L Holman CD Prevalence of occupational exposures and protective practices in Australian female veterinarians Aust Vet J 2007 85 1-2 32 38 10.1111/j.1751-0813.2006.00077.x 17300451

[B6] Wiggins P Schenker MB Green R Samuels S Prevalence of hazardous exposures in veterinary practice Am J Ind Med 1989 16 1 55 66 10.1002/ajim.4700160107 2750751

[B7] Wilkins JR Bowman ME Needlestick injuries among female veterinarians: frequency, syringe contents and side-effects Occup Med (Lond) 1997 47 8 451 457 10.1093/occmed/47.8.451 9604476

[B8] Trimpop R Kirkcaldy B Athanasou J Cooper C Individual differences in working hours, work perceptions and accident rates in veterinary surgeries Work Stress 2000 14 2 181 188 10.1080/026783700750051685

[B9] Wilkins MJ Bartlett PC Judge LJ Erskine RJ Boulton ML Kaneene JB Veterinarian injuries associated with bovine TB testing livestock in Michigan, 2001 Prev Vet Med 2009 89 3-4 185 190 10.1016/j.prevetmed.2009.02.014 19303154

[B10] Smith DR Leggat PA Speare R Musculoskeletal disorders and psychosocial risk factors among veterinarians in Queensland, Australia Aust Vet J 2009 87 7 260 265 10.1111/j.1751-0813.2009.00435.x 19573148

[B11] Survey details stress factors that influence Australian vets Aust Vet J 2002 80 9 522 524

[B12] Gardner DH Hini D Work-related stress in the veterinary profession in New Zealand N Z Vet J 2006 54 3 119 124 16751842 10.1080/00480169.2006.36623

[B13] Milani M Practitioner-client communication: when goals conflict Can Vet J 2003 44 8 675 678 13677603 PMC340246

[B14] Milani M Problematic client-veterinarian relationships: the "yes, buts" Can Vet J 2006 47 10 1025 1028 17078255 PMC1571120

[B15] Milani M Nothing to fear, but ... Part II: The clinician and fears of emotional trauma Can Vet J 2007 48 6 596 599 17616055 PMC1876185

[B16] Faragher T Suicide in Australian veterinarians Aust Vet J 2008 86 7 249 10.1111/j.1751-0813.2008.00323.x 18616473

[B17] Jones-Fairnie H Ferroni P Silburn S Lawrence D Suicide in Australian veterinarians Aust Vet J 2008 86 4 114 116 10.1111/j.1751-0813.2008.00277.x 18363981

[B18] Meehan MP Bradley L Identifying and evaluating job stress within the Australian small animal veterinary profession Aust Vet Practit 2007 37 2 70 83

[B19] Heath TJ Longitudinal study of veterinarians from entry to the veterinary course to 10 years after graduation: attitudes to work, career and profession Aust Vet J 2002 80 8 474 478 10.1111/j.1751-0813.2002.tb12468.x 12224615

[B20] Harling M Strehmel P Schablon A Nienhaus A Psychosocial stress, demoralization and the consumption of tobacco, alcohol and medical drugs by veterinarians J Occup Med Toxicol 2009 4 1 4 10.1186/1745-6673-4-4 19243579 PMC2651184

[B21] Leggat PA Smith DR Speare R Exposure rate of needlestick and sharps injuries among Australian veterinarians J Occup Med Toxicol 2009 4 1 25 10.1186/1745-6673-4-25 19712488 PMC2744915

[B22] Likert R A technique for the measurement of attitudes Arch Psychol 1932 140 1 55

[B23] Bland JM Altman DG Cronbach's alpha BMJ 1997 314 7080 572 9055718 10.1136/bmj.314.7080.572 PMC2126061

[B24] Cronbach LJ Coefficient alpha and the internal structure of tests Psychometrika 1951 16 3 297 334 10.1007/BF02310555

[B25] Heath TJ Number and distribution of Australian veterinarians in 1991 and 2001 Aust Vet J 1981 80 7 400 405 10.1111/j.1751-0813.2002.tb10995.x 12222599

[B26] Gamperiene M Nygard JF Sandanger I Waersted M Bruusgaard D The impact of psychosocial and organizational working conditions on the mental health of female cleaning personnel in Norway J Occup Med Toxicol 2006 1 1 24 10.1186/1745-6673-1-24 17078871 PMC1636641

[B27] Heath TJ Longitudinal study of career plans and directions of veterinary students and recent graduates during the first five years after graduation Aust Vet J 1998 76 3 181 186 10.1111/j.1751-0813.1998.tb10125.x 9578754

[B28] Rovik JO Tyssen R Hem E Gude T Ekeberg O Moum T Vaglum P Job stress in young physicians with an emphasis on the work-home interface: a nine-year, nationwide and longitudinal study of its course and predictors Ind Health 2007 45 5 662 671 10.2486/indhealth.45.662 18057809

[B29] Rohlf V Bennett P Perpetration-induced traumatic stress in persons who euthanize nonhuman animals in surgeries, animal shelters, and laboratories Soc Anim 2005 13 3 201 219 10.1163/1568530054927753 16270455

[B30] Smith DR Wei N Zhang YJ Wang RS Musculoskeletal complaints and psychosocial risk factors among physicians in mainland China Int J Ind Ergon 2006 36 6 599 603 10.1016/j.ergon.2006.01.014

[B31] Tyssen R Health problems and the use of health services among physicians: a review article with particular emphasis on Norwegian studies Ind Health 2007 45 5 599 610 10.2486/indhealth.45.599 18057803

[B32] Gamperiene M Nygard JF Sandanger I Lau B Bruusgaard D Self-reported work ability of Norwegian women in relation to physical and mental health, and to the work environment J Occup Med Toxicol 2008 3 1 8 10.1186/1745-6673-3-8 18430207 PMC2373783

[B33] Vets need to learn anti-stress skills Aust Vet J 2002 80 9 522

[B34] Heath TJ Recent veterinary graduates over the last five decades: initial career experiences Aust Vet J 2005 83 10 626 632 10.1111/j.1751-0813.2005.tb13276.x 16255287

[B35] Edimansyah B Rusli B Naing L Effects of short duration stress management training on self-perceived depression, anxiety and stress in male automotive assembly workers: a quasi-experimental study J Occup Med Toxicol 2008 3 1 28 10.1186/1745-6673-3-28 19021918 PMC2600780

[B36] Whitten L Occupational hazards in veterinary practice J Occup Health Safety - Aust NZ 1989 5 6 523 526

[B37] Edwards P Roberts I Clarke M DiGuiseppi C Pratap S Wentz R Kwan I Increasing response rates to postal questionnaires: systematic review BMJ 2002 324 7347 1183 10.1136/bmj.324.7347.1183 12016181 PMC111107

[B38] Wilkins JR Hueston WD Crawford JM Steele LL Gerken DF Mixed-mode survey of female veterinarians yields high response rate Occup Med (Lond) 1997 47 8 458 462 10.1093/occmed/47.8.458 9604477

